# Does quality of life among breast cancer survivors one year after diagnosis differ depending on urban and non-urban residence? A comparative study

**DOI:** 10.1186/1477-7525-8-3

**Published:** 2010-01-07

**Authors:** Tracey DiSipio, Sandi C Hayes, Beth Newman, Joanne Aitken, Monika Janda

**Affiliations:** 1 School of Public Health, Institute of Health and Biomedical Innovation, Queensland University of Technology, Victoria Park Road, Kelvin Grove, Queensland, 4059, Australia; 2 Viertel Centre for Research in Cancer Control, Cancer Council Queensland, PO Box 201, Spring Hill, Queensland, 4004, Australia

## Abstract

**Background:**

This study examined the quality of life (QOL), measured by the Functional Assessment of Cancer Therapy (FACT) questionnaire, among urban (n = 277) and non-urban (n = 323) breast cancer survivors and women from the general population (n = 1140) in Queensland, Australia.

**Methods:**

Population-based samples of breast cancer survivors aged < 75 years who were 12 months post-diagnosis and similarly-aged women from the general population were recruited between 2002 and 2007.

**Results:**

Age-adjusted QOL among urban and non-urban breast cancer survivors was similar, although QOL related to breast cancer concerns was the weakest domain and was lower among non-urban survivors than their urban counterparts (36.8 versus 40.4, *P *< 0.01). Irrespective of residence, breast cancer survivors, on average, reported comparable scores on most QOL scales as their general population peers, although physical well-being was significantly lower among non-urban survivors (versus the general population, *P *< 0.01). Overall, around 20%-33% of survivors experienced lower QOL than peers without the disease. The odds of reporting QOL below normative levels were increased more than two-fold for those who experienced complications following surgery, reported upper-body problems, had higher perceived stress levels and/or a poor perception of handling stress (*P *< 0.01 for all).

**Conclusions:**

Results can be used to identify subgroups of women at risk of low QOL and to inform components of tailored recovery interventions to optimize QOL for these women following cancer treatment.

## Background

Breast cancer is a major public health concern, with one in eight women developing the disease before the age of 85 years in developed countries of the world [1,2]. Despite therapeutic advances, which have contributed to improvements in survival (five-year survival currently 87%) [3] women continue to experience considerable physical and psychosocial dysfunction during and following treatment. While these quality of life (QOL) concerns are short-lived for some, others may struggle to regain expected levels of QOL longer term.

QOL has been associated with adherence to treatment [4] and prognosis [5,6] and is now recognized as an important research outcome. International research on factors that influence QOL among breast cancer survivors has been extensive (over 300 published studies in 2008 alone integrated QOL as an outcome). Socio-demographic (e.g., income), general health (e.g., medical conditions) and treatment (e.g., adjuvant therapy) characteristics each have been associated with QOL [7], with the strength and consistency of the associations dependent on the characteristic of interest. Nevertheless, there remain subgroups of women for whom limited information on QOL is available, including those women who reside outside major metropolitan areas. This is important because approximately one-third of new breast cancer cases live outside major metropolitan areas [2].

In Australia, geographic residence influences stage at diagnosis and type of surgery, with those living in rural areas more likely to have a mastectomy than their urban counterparts (38% versus 25%, respectively) [8-10]. Geographic residence also influences access to health services [11], as fewer than half of regional/rural hospitals administer chemotherapy [12], and fewer still provide radiotherapy services [13]. Further, rural Australian women often have to travel in excess of 100 kilometers (i.e., 62 miles) to receive adjuvant treatment and are away from home for approximately 20 to 43 days for chemotherapy and radiotherapy treatment, respectively [14,15]. Hence, it seems plausible that rural women with breast cancer may have unique and additional burdens, such as disruption to family life, work and financial security [14,16], which ultimately may influence QOL differently to that observed for women residing in urban areas.

Research that compares QOL between urban and non-urban cancer survivors is lacking, and from those studies that exist, results are inconsistent. Two studies suggest that rural breast cancer survivors fare worse [17,18], while one indicates that QOL is superior among a rural group of mixed cancer survivors [19], when compared with their urban counterparts. Further, there is a paucity of information comparing the QOL among cancer groups with that of the general population, making interpretation of findings challenging. Therefore, this paper examines whether QOL differs between urban and non-urban women 12 months following breast cancer diagnosis and compares their QOL with women from the general population residing in their respective geographic areas. We also sought to identify characteristics of breast cancer survivors associated with reporting QOL below normative levels.

## Methods

### Breast cancer study samples

The Pulling Through Study (PTS) was a longitudinal, population-based study among breast cancer survivors living within 100 kilometers (i.e., 62 miles) of the capital city of Brisbane in Queensland, Australia, and diagnosed in 2002 [20,21]. This study was extended to include survivors from non-urban areas of Queensland, diagnosed between April 2006 and March 2007 [22]. The Accessibility/Remoteness Index of Australia (ARIA+) classification system was used to define place of residence as either major city, inner regional, outer regional, remote or very remote, and is based on road distance and population size of the nearest town [23]. The selected localities within the perimeter of Brisbane fall within the ARIA+ classification for major cities and hereafter are referred to as 'urban'. Residents of inner regional, remote and very remote areas were pooled as the 'non-urban' group and reflect the reduced access to a range of oncology services experienced by those who live outside state capital cities, irrespective of the level of remoteness [12].

Eligible women, diagnosed with unilateral breast cancer at age 74 years or younger, were randomly selected through the Queensland Cancer Registry (target sample). All cancer diagnoses in Queensland are required to be reported to the Registry and therefore these records provide an accurate sampling frame for recruitment. Since breast cancer is mostly a disease of women 50 yrs or older and to ensure adequate numbers were available for specific age group analyses, younger women were over-sampled in the urban arm of the study, while 100% of eligible non-urban women were recruited for all age groups. Following appropriate ethical approval and the requirements of the cancer registry, doctor consent to contact eligible women (provided for 82% of the urban sample and 90% of the non-urban sample) and participant consent was sought. Overall, 277 urban and 323 non-urban women returned completed quuestionnaires at 12 months post-diagnosis (66% and 71% of eligible women with doctor consent for the urban and non-urban arms, respectively).

### General population study sample

Following ethical approval, the general Queensland population sample was derived from the Queensland Cancer Risk Study (QCRS), a population-based survey conducted in 2004 among English-speaking residents of Queensland, aged 20-75 years, randomly sampled within strata defined by gender, age and geographic region (defined by the ARIA+ classification as as major city, inner regional, outer regional or remote/very remote). Further details about the study methods are described elsewhere [24]. Briefly, of the 8,398 adults who agreed to participate in the self-administered questionnaire, 5822 (69.3%) returned surveys, of which 2727 contained QOL information. Analyses reported in this paper include women for whom QOL data were available and who had no prior history of breast cancer, with 675 living in urban and 465 in non-urban areas of Queensland, as defined by the ARIA+.

### Questionnaires

QOL was measured among women with breast cancer at 12 months post-diagnosis using the Functional Assessment of Cancer Therapy (FACT-G) questionnaire, which is comprised of 27 items rated on a five-point Likert scale (ranging from 0 = 'not at all' to 4 = 'very much') and includes four subscales (physical, social, emotional, and functional well-being). Higher scores represent better well-being. Women in the QCRS received the general population FACT instrument (FACT-GP), which is identical to the FACT-G except it excludes six illness-related items inappropriate for the general population [25,26]. Overall FACT-GP summary scores and subscales were pro-rated as per the FACT manual to obtain scores comparable to the FACT-G [27], resulting in total scores for all study groups ranging from 0-108 for overall QOL, 0-28 for the physical, social, and functional well-being subscales, and 0-24 for the emotional well-being subscale. Women with breast cancer also completed 13 questions on breast cancer concerns and arm morbidity (FACT-B+4), with total scores from 0-52 for the breast cancer concerns subscale, and 0-160 for overall FACT-B+4. The FACT instrument has excellent reliability and validity [28].

Demographic (age, marital status, educational level, private health insurance, occupation [29,30] and income), general health (smoking status, body mass index, co-morbidities, complications following surgery, upper-body function [31], physical activity and stress levels including perceived handling of stress) and treatment (chemotherapy, radiotherapy, hormone therapy) characteristics for the breast cancer study participants were also obtained via the questionnaire, whereas information on tumor characteristics were abstracted from histopathology reports (e.g., type of surgery, maximum tumor size and grade, and lymph node status).

### Statistical analysis

Distributions of the FACT scores were approximately normal and hence were summarized as means with 95% confidence intervals (CIs) using SPSS (SPSS Inc, Chicago, IL, version 14). Analysis of variance tests compared age-adjusted mean QOL scores at 12 months post-diagnosis between urban and non-urban breast cancer survivors. Comparisons between breast cancer survivors and women from the general population involved general linear regression models to obtain QOL scores adjusted for characteristics that differed between the groups (i.e., potential confounding factors).

Descriptive results presented in this study have been adjusted for the sampling fraction used to identify younger breast cancer patients from urban areas (weighting applied: < 50 years:1.0; ≥50 years:1.3). The general population comparison group was also weighted by age, based on Australian Bureau of Statistics data, so that results reflect the actual female Queensland resident population (weighting applied for urban, regional, outer regional, remote and very remote: < 50 years:1.3, 1.3, 1.4, 1.5 and 0.9, respectively; ≥50 years:0.8, 0.8, 0.7, 0.6 and 1.1, respectively) [33]. The conventional *P *< 0.05 level (two-tailed) was accepted as statistically significant. Differences of eight or more points in mean FACT-B+4 scores, five or more points in mean FACT-G scores, three or more points on the breast cancer concerns subscale and two or more points for all other subscales between urban and non-urban breast cancer survivors or between women with breast cancer and their general population peers were considered clinically important, as recommended by developers of the FACT [25]. For correlates, a difference in odds ratios (ORs) of ≥1.8 or ≤0.6 was considered to be of potential clinical relevance.

As suggested by Fayers [34], a new outcome measure was calculated to characterize breast cancer survivors whose QOL was below normative levels. QOL values were calculated for each five-year age stratum of the general population study group and subtracted from the QOL score within the same age group of women with breast cancer (i.e., case FACT-G minus general population comparison group FACT-G) separately by urban and non-urban residence [34]. Positive scores indicate higher QOL, and negative scores indicate lower QOL, among cases relative to age- (within five years) and residence-matched peers. Relative overall QOL (FACT-G) was then categorized into groups using score differentials considered clinically important to investigate the proportions of breast cancer survivors with relative overall QOL lower than (-5.0 points or more), similar to (>-5.0 to < +5.0) or better than (+5.0 points or more) the general population study group. Relative QOL was also calculated for each subscale, using two points as the critical threshold. A dichotomous outcome variable was defined, combining the 'similar' and 'better' groups, and binary logistic regression was used to generate ORs and 95% CIs to identify demographic, general health, and clinical characteristics associated with QOL status below the norm compared to the 'similar/better' group. A range of potentially important correlates were explored, however, only those that were found to be statistically significant or clinically important are reported. Formal tests of interactions between residence and each of the characteristics of interest did not yield any statistically significant results, therefore pooled results, adjusted for residence, are presented.

## Results

### Sample characteristics

Demographic and disease characteristics were similar for the women with breast cancer in this study and those in the target sample. The majority of women (75-80%) were diagnosed with infiltrating ductal carcinoma, approximately 60% received complete local excision of their tumour and more than 50% had 10 or more lymph nodes removed. However, participants among urban breast cancer survivors had somewhat smaller tumor size (median tumour size was 14 mm) when compared with the target sample [20-22]. For the majority of demographic and general health characteristics, women with breast cancer had similar characteristics, irrespective of place of residence. However, the urban compared to the non-urban breast cancer sample was more likely to be unmarried, have private health insurance and report fewer co-morbidities, and less likely to be obese (Table 1). Non-urban compared to urban women with breast cancer were more likely to have multiple forms of adjuvant therapy and less likely to report multiple complications.

**Table 1:** Participant Characteristics

Characteristics		Urban general population^a^(n = 675)		Urban breast cancer survivors^b ^(n = 277)		Non-urban general population^a ^(n = 465)		Non-urban breast cancer survivors (n = 323)	
				
		n	%	n	%	n	%	n	%
**Demographic characteristics**									
Age (years)					*				
<50		298	57.0	99	30.0	185	55.6	107	33.1
50+		377	43.0	178	70.0	280	44.4	216	66.9
Marital status					*				†
Married, or living as married		520	77.6	186	66.6	361	80.1	248	76.8
Not married		155	22.4	91	33.4	104	19.9	75	23.2
Education level					*				*
Grade 10 or below		219	30.2	125	46.5	186	37.0	173	53.6
Grade 12/Trade/TAFE		278	42.5	95	33.7	183	40.7	102	31.6
University or college degree		178	27.3	57	19.9	96	22.3	48	14.9
Private health insurance status					*				†
Yes		398	58.0	200	72.4	222	48.5	140	43.3
No		277	42.0	77	27.6	243	51.5	183	56.7
**General health characteristics**									
Smoking status									
Never smoked		364	53.6	163	59.3	250	52.9	184	57.0
Past smoker		216	31.2	84	30.0	149	31.7	102	31.6
Current smoker		95	15.2	30	10.7	66	15.3	37	11.5
Physical activity^c^									*
Sedentary		94	17.5	34	12.6	81	13.7	68	21.1
Insufficient activity		194	27.9	67	23.9	125	29.4	55	17.0
Sufficient activity		387	54.6	176	63.5	259	57.0	200	61.9
Body mass index (kg/m^2^)					*				†
Underweight/Normal (up to 24.9)		335	41.5	108	38.5	183	50.7	116	35.9
Overweight (25-29.9)		203	27.4	80	29.0	134	29.6	91	28.2
Obese (30+)		106	25.1	55	20.0	119	14.9	100	31.0
Missing		31	6.0	34	12.5	29	4.7	16	5.0
Number of co-morbidities^d^					*				*^†^
None		126	21.6	68	23.8	84	21.4	55	17.0
One		158	19.0	68	23.7	79	24.7	74	22.9
Two		133	19.7	69	25.6	95	19.2	79	24.5
Three or more		258	39.6	72	27.0	206	34.7	115	35.6
**Clinical characteristics**									
Adjuvant treatment		-	-			-	-		†
None				42	15.8			59	18.3
Chemotherapy only				34	11.3			33	10.2
Radiotherapy only				119	43.8			105	32.5
Both				82	29.1			126	39.0
Number of complications^e^		-	-			-	-		†
None				54	19.7			133	41.2
Yes, one to four				223	80.3			190	58.8

*Abbreviations*:

* Statistically significant difference (*P *< 0.05) between the general population and breast cancer survivors by place of location.

† Statistically significant difference (*P *< 0.05) between urban and non-urban breast cancer survivors.

*Notes*:

(a) Column percentages are standardized to the 2003 Queensland population by age.

(b) Column percentages have been weighted to correct for sampling.

(c) 'Sedentary' is defined as no activity; 'Insufficient' time is defined as participating in some activity but less than 150 minutes per week, using the sum of walking, moderate activity and vigorous activity (weighted by 2); 'Sufficient' time is defined as 150 minutes per week, using the sum of walking, moderate activity and vigorous activity (weighted by 2) [45].

(d) Co-morbidities include heart conditions, high blood pressure, high cholesterol, stroke, diabetes, lung conditions, stomach or duodenal ulcer, migraine or headaches, arthritis, cancer other than breast, depression and other prolonged or serious illness.

(e) Complications include wound infection, other infection, skin reaction, seroma.

A comparison of women with or without breast cancer showed significant differences for several demographic and general health characteristics (Table 1). Breast cancer survivors tended to be older or have lower educational levels when compared with the general population, irrespective of residence. In addition, urban breast cancer survivors were more likely to be single, have private health insurance, and/or fewer co-morbidities (other than breast cancer), while non-urban breast cancer survivors were more likely to be sedentary and/or have two or more co-morbidities (other than breast cancer), when compared with their general population counterparts. While there was a significant (*P *< 0.05) difference in body mass index between urban breast cancer survivors and their general population peers, this was attenuated when missing values were omitted from analyses.

### QOL among urban and non-urban breast cancer survivors

Although urban breast cancer survivors reported higher age-adjusted QOL summary and subscale scores than their non-urban counterparts 12 months following diagnosis, differences did not reach the threshold for clinical importance even for those subscales that were statistically significant (physical, emotional, and overall QOL, *P *< 0.01). In contrast, well-being related to breast cancer concerns was lower among non-urban compared to urban survivors by statistical (*P *< 0.01) and clinical criteria (Table 2). Furthermore, for both groups, women reported most detriment to their QOL for this subscale, with participants reporting mean values below 80% of the maximum score on average. Participants reported mean values at approximately 80% of the maximum score for all other subscales.

**Table 2:** QOL scores at 12 months post-diagnosis for urban and non-urban breast cancer survivors

Quality of life		Urban breast cancer survivors (n = 277)		Non-urban breast cancer survivors (n = 323)		Differences between residence groups	
			
		Mean^a^	95% CI	Mean^a^	95% CI	*P-*Value	clinical^b^
Physical well-being (0-28)		24.7	24.1, 25.2	22.8	22.3, 23.3	< 0.01	✗
Social well-being (0-28)		22.7	22.0, 23.4	22.5	21.8, 23.1	0.67	✗
Emotional well-being (0-24)		20.1	19.6, 20.5	19.2	18.7, 19.6	< 0.01	✗
Functional well-being (0-28)		22.4	21.8, 23.1	21.7	21.1, 22.3	0.09	✗
Breast cancer concerns (0-52)		40.4	39.5, 41.4	36.8	36.0, 37.7	< 0.01	✓
FACT-G (0-108)		89.7	87.9, 91.5	86.3	84.7, 88.0	< 0.01	✗
FACT-B+4 (0-160)		130.2	127.7, 132.7	122.6	120.3, 125.0	< 0.01	✗

*Abbreviations*:

FACT-G: Functional Assessment of Cancer Therapy-General; FACTB+4: Functional Assessment of Cancer Therapy-Breast additional four questions.

*Notes*:

(a) Adjusted for age.

(b) ✗: clinically meaningful difference between groups (two+ points for physical, social, emotional and functional well-being, three+ points for breast cancer concerns, five+ points for FACT-G, eight+ points for FACT-B+4); ✓: no clinically meaningful difference between groups.

### Breast cancer survivors' QOL compared to the general population

Table 3 presents the subscale and overall mean FACT-G scores for breast cancer survivors compared with women from the general population, stratified by residence and adjusted for potential confounding factors. At 12 months post-diagnosis, urban and non-urban breast cancer survivors reported clinically higher social well-being compared with their general population peers, and non-urban breast cancer survivors also reported clinically lower physical well-being (*P *< 0.01 for all). Scores for emotional, functional and overall (FACT-G) QOL were clinically comparable to their counterparts from the general population despite a statistically significant difference for emotional and functional well-being (*P *< 0.01).

**Table 3:** Adjusted mean QOL for women with breast cancer compared with the general population stratified by residence location

Quality of life		Residence	General population		Breast cancer survivors		Difference between groups	
			
			Mean^a^	95% CI	Mean^a^	95% CI	*P*-Value	clinical^b^
Physical well-being (0-28)		urban	25.0	24.8, 25.3	24.2	23.8, 24.6	< 0.01	✗
		non-urban	25.1	24.7, 25.5	22.7	22.2, 23.2	< 0.01	✓
Social well-being (0-28)		urban	19.9	19.5, 20.4	22.4	21.6, 23.2	< 0.01	✓
		non-urban	19.6	19.0, 20.2	22.4	21.7, 23.1	< 0.01	✓
Emotional well-being (0-24)		urban	21.1	20.8, 21.4	19.6	19.1, 20.0	< 0.01	✗
		non-urban	20.9	20.5, 21.2	19.2	18.8, 19.7	< 0.01	✗
Functional well-being (0-28)		urban	20.6	20.2, 21.1	22.0	21.3, 22.7	< 0.01	✗
		non-urban	20.2	19.7, 20.8	21.6	21.0, 22.3	< 0.01	✗
FACT-G (0-108)		urban	86.9	85.8, 88.0	88.0	86.3, 89.8	0.28	✗
		non-urban	85.8	84.4, 87.3	86.2	84.4, 87.9	0.79	✗

*Abbreviations*:

FACT-G: Functional Assessment of Cancer Therapy-General.

*Notes*:

(a) Adjusted for age (years), marital status (married or living as married, not married), education level (low, moderate, high), private health insurance status (yes, no), smoking status (never smoked, past smoker, current smoker), physical activity (sedentary, insufficient, sufficient), body mass index (underweight/healthy, overweight, obese, missing), and co-morbidities (none, one, two, three or more).

(b) ✗: clinically meaningful difference between groups (two+ points for physical, social, emotional and functional well-being, five+ points for FACT-G); ✓: no clinically meaningful difference between groups.

**Figure 1:**
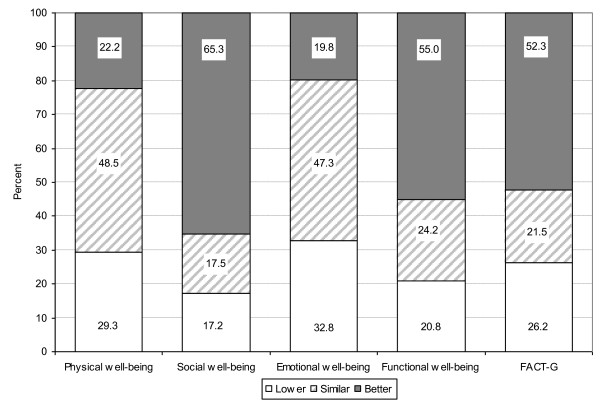
**Proportions of breast cancer survivors whose relative QOL at 12 months post-diagnosis was lower than, similar to, or better than general population peers**.

Using the new outcome measure of QOL relative to age and residency-matched women from the general population, depending on the specific QOL scale, between 17.2% and 32.8% of all women with breast cancer reported clinically *lower *QOL 12 months following diagnosis than age- (within five years) and residence-matched women without the disease. A further 17.5%-48.5% of women reported similar QOL, while the remainder (19.8%-65.3%) reported clinically better QOL (Figure 1). The subscales with the highest proportions below the norm were emotional (32.8%) and physical (29.3%) well-being, and overall QOL (26.2%).

### Characteristics associated with QOL below normative levels among breast cancer survivors

Following adjustment for potential confounding factors, a range of characteristics were associated with breast cancer survivors reporting overall (FACT-G) QOL below normative levels (Table 4), but place of residence (i.e., urban versus non-urban) was not one of these (Odds Ratio (OR) = 1.06; 95% Confidence interval (CI) = 0.64-1.74). Experiencing one or more complications following surgery was associated with two-fold increased odds (OR = 2.26, 95% CI = 1.31-3.90; *P *< 0.01) of reporting reduced QOL, while upper-body function below the median, moderate or higher stress levels and poor perceived handling of stress were each associated with at least four-fold increased odds of reporting reduced QOL (ORs ranging from 4.24-4.77, *P *< 0.01, see Table 4). A marker of higher socioeconomic status, having private health insurance, was associated with a 0.6 odds of reporting lower relative QOL (95% CI = 0.37-0.99, *P *= 0.05).

**Table 4:** Correlates of QOL (FACT-G) below the norm at 12 months post-diagnosis among breast cancer survivors^a^

Characteristics	Model			
	
	n	OR^b^	95% CI	*P-*Value
Place of residence				0.82
Urban	277	1.00	-	
Non-urban	323	1.06	0.64, 1.74	
Age (years)				0.21
<50	205	1.00	-	
50+	395	0.72	0.43, 1.20	
Occupation				0.38
Professional	182	1.00	-	
White-collar worker	176	1.16	0.64, 2.11	
Blue-collar worker	31	2.53	0.95, 6.76	
Homemaker	106	1.29	0.63, 2.62	
Retired/student	105	0.92	0.43, 1.96	
Yearly income				0.15
<$52,000	336	1.00	-	
$52,000+	207	0.60	0.35, 1.04	
Missing	57	0.66	0.28, 1.55	
Private health insurance status				0.05
No	217	1.00	-	
Yes	383	0.61	0.37, 0.99	
Overall histological grade				0.34
Grade 1	142	1.00	-	
Grade 2	219	0.58	0.32, 1.06	
Grade 3	208	0.69	0.38, 1.24	
Not available	31	0.54	0.14, 2.10	
Number of complications^c^				<0.01
None	187	1.00	-	
Yes, one to four	413	2.26	1.31, 3.90	
Upper-body function				<0.01
Good function (<11)	301	1.00	-	
Poor function (11+)	258	4.44	2.66, 7.40	
Missing	41	3.63	1.45, 9.07	
Amount of stress				<0.01
Very little/some	371	1.00	-	
A moderate amount/a lot	229	4.77	2.93, 7.76	
Perceived handling of stress				<0.01
Very well/fairly well	526	1.00	-	
Not well/not well at all	74	4.24	2.21, 8.15	

*Notes*:

(a) Mutually adjusted for all variables in the model.

(b) Odds ratio for QOL below the norm (R^2 ^= 0.43).

(c) Complications include wound infection, other infection, skin reaction, seroma.

## Discussion

Urban and non-urban breast cancer survivors reported similar levels of QOL 12 months following diagnosis, overall and for subscales. The sole exception was the breast cancer concerns subscale, which showed that non-urban residents fared worse than their urban counterparts. When comparing breast cancer survivors to age- and residence-matched peers, the only detriment to QOL was among non-urban breast cancer survivors who reported statistically and clinically poorer physical well-being. Overall, up to one in three breast cancer survivors reported QOL below the age- and residency-matched general female population. The major independent correlates of reporting overall QOL below that of age-matched women without breast cancer were complications following surgery, poorer upper-body function, higher perceived stress levels, and poor perception of handling stress.

Despite the known differences by geographic residence with regards to access to services, availability of treatment and survival outcomes, our results indicate only minor disparities in QOL between urban and non-urban breast cancer survivors 12 months post-diagnosis. The subscale measuring breast cancer-specific concerns yielded the lowest values (based on percent of maximum score) reported by all survivors, but in particular for women living in non-urban areas. Items within this subscale deal with treatment-related symptoms, such as swelling of the arms, pain, shortness of breath, body image and sexuality. These results support existing research which demonstrates that while QOL among breast cancer survivors improves considerably during the first year following completion of treatment, breast cancer treatment-related concerns (such as arm dysfunction, poor body image, and sexual dysfunction) may persist [35-39].

It is plausible that non-urban survivors suffer in terms of their breast cancer-specific QOL, more so than urban survivors, as a consequence of inequalities in accessing specialised services. However, study-specific data collection procedures may also have contributed. QOL scores were derived from the third questionnaire for participants in the longitudinal urban breast cancer study, whereas the first (and only) questionnaire was the source of QOL data for non-urban breast cancer participants. Therefore urban survivors may have responded differently to QOL questions over time, not only because their QOL changed, but also because they may have become used to answering questions about QOL and might have over time changed their perception of QOL. This response shift may, in part, explain what appears to be a more positive breast cancer-specific QOL among urban survivors than non-urban survivors. However, the difference in QOL was observed on most but not all subscales, suggesting that response shift played a minor role in our findings.

On average, QOL was similar for breast cancer survivors and general population peers, for both urban and non-urban residents, similar to results reported by other authors studying QOL among breast cancer survivors 12 months [40,41] or longer [18,26,42] following diagnosis. The high FACT-G scores observed among breast cancer survivors are somewhat surprising, because patients frequently report ongoing symptoms and long-term side-effects [35,36,39]. High functional and social well-being reported by breast cancer survivors compared to their general population counterparts contributed to their overall high FACT-G score and contradicts previous research [18,26,40-42]. However, the literature is dominated by studies using the European Organisation for the Research and Treatment of Cancer QOL questionnaire (EORTC QLQ-C30) [18,26,40,41]. The social well-being subscale of the EORTC QLQ-C30 and the FACT have been shown to be poorly correlated (r = 0.09) [43] suggesting they measure different aspects of social well-being. Furthermore, QOL domains measured by the FACT-G may be more relevant to short-term recovery. Whereas 12 months or longer after diagnosis, alternate issues may become more important for QOL, such as fear of recurrence or making meaning of the cancer experience. More recently, survivorship-specific QOL instruments have been developed, and further research is needed to assess whether these will uncover additional medium- to long-term survivorship issues [44].

Despite overall QOL similarities between survivors and their general population peers, up to one-third (depending on the subscale) of survivors continued to experience lower QOL 12 months following diagnosis of breast cancer. To our knowledge, despite Fayers suggesting advanced analytical procedures using normative scores in 2000 [34], this is the first study to assess correlates of lower QOL among breast cancer survivors in this manner. The results demonstrate that experiencing one or more treatment-related complications, reporting lower upper-body function than the median, moderate to high stress levels and/or perceived poor handling of stress could reduce the odds of good QOL two- to four-fold. The cross-sectional nature of the data denotes that these characteristics are correlates of QOL but not necessarily causes. Moreover, the relative QOL index used to identify these correlates may be focusing on those women with breast cancer who would have been in the lower part of the QOL range even before they had the disease. Regardless, these correlates have relevance for identifying subgroups of breast cancer survivors who require assistance to regain QOL to levels expected among age-matched peers from the general population.

Several key design features of this work highlight the strength and importance of the findings. Results were obtained from population-based urban and non-urban breast cancer samples, representative of their respective target populations [20-22], and therefore results are likely generalizable to the wider population of breast cancer survivors. Further, QOL of survivors were compared to peers without breast cancer, including matching for place of residence, allowing for more accurate interpretation of meaning of results. At a glance, the results from this study suggest that, overall, women with breast cancer fare well by 12 months following diagnosis; however, interventions are needed to improve breast cancer-related concerns among all women with breast cancer and physical well-being among non-urban survivors. These should specifically recruit those survivors who experience complications following surgery, upper-body dysfunction and/or those with a greater burden of stress (i.e., higher amounts and/or poor self-perceived handling of stress). Interventions that address such concerns and that are accessible for all women, irrespective of place of residence, may help facilitate a faster return to optimal QOL in the future.

## Conclusions

Overall, the QOL of breast cancer survivors living in rural and urban areas was similar except for breast cancer related concerns being more dominant in women from rural locations. Among all women about 20%-33% have lower QOL one year past diagnosis compared to age matched women from the general population without breast cancer and thus could benefit from additional support and interventions.

## List of abbreviations

ARIA+: Accessibility/Remoteness Index of Australia; CI: Confidence Interval; EORTC: European Organisation for the Research and Treatment of Cancer; FACT: Functional Assessment of Cancer Therapy; FACT-G: Functional Assessment of Cancer Therapy-General; FACT-GP: Functional Assessment of Cancer Therapy-General Population; FACTB+4: Functional Assessment of Cancer Therapy-Breast additional four questions; OR: Odds Ratio; PTS: Pulling Through Study; QOL: Quality of Life; QCRS: Queensland Cancer Risk Study.

## Competing interests

The authors declare that they have no competing interests.

## Authors' contributions

TD carried out data collection and analysis. SH, BN, and MJ supervised TD and contributed to data interpretation and manuscript writing. JA supervised data collection at the Cancer Registry and provided critical input in data collection, analysis and manuscript. All authors read and approved the final manuscript.
